# Association of *ENPP1 *gene polymorphisms with hand osteoarthritis in a Chuvasha population

**DOI:** 10.1186/ar1786

**Published:** 2005-07-13

**Authors:** Eun-Kyung Suk, Ida Malkin, Stefan Dahm, Leonid Kalichman, Nico Ruf, Eugene Kobyliansky, Mohammad Toliat, Frank Rutsch, Peter Nürnberg, Gregory Livshits

**Affiliations:** 1 Gene Mapping Center, Max Delbrück Center for Molecular Medicine, Berlin, Germany; 2 Human Population Biology, Research Unit, Department of Anatomy and Anthropology, Sackler Faculty of Medicine, Tel Aviv University, Ramat Aviv, Tel Aviv, Israel; 3 Bioinformatics Section, Max Delbrück Center for Molecular Medicine, Berlin, Germany; 4 Department of Pediatrics, University Medical School Münster, Germany; 5 Institute of Medical Genetics, Charité – University Hospitals of Berlin, Germany; 6 Cologne Center for Genomics and Institute for Genetics, University of Cologne, Germany

## Abstract

Periarticular calcification is a common attendant symptom of generalized arterial calcification of infancy, a rare Mendelian disorder caused by mutations of the gene coding for ectonucleotide pyrophosphatase/phosphodiesterase 1 (ENPP1). This prompted us to perform a family-based association study to test the hypothesis that genetic variation at the *ENPP1 *locus is involved in the etiology of osteoarthritis of the hand. The study population comprised 126 nuclear families with 574 adult individuals living in small villages in the Chuvasha and Bashkirostan autonomies of the Russian Federation. The extent of osteoarthritis was determined by analyzing plain hand radiographs. The outcome of a principal component analysis of osteoarthritis scores of a total of 28 joints of both hands was used as a primary phenotype in this study. Maximum likelihood estimates of the variance component analysis revealed a substantial contribution of genetic factors to the overall trait variance of about 25% in this homogeneous population. Three short tandem repeat (STR) polymorphisms – one intragenic and two flanking markers – and four single-nucleotide polymorphisms were tested. The markers tagged the *ENPP1 *locus at nearly equal intervals. We used three different transmission disequilibrium tests and obtained highly significant association signals. Alleles of the upstream microsatellite marker as well as several single-nucleotide polymorphism haplotypes consistently revealed the association. Thus, our data highlights variability of *ENPP1 *as an important genetic factor in the pathogenesis of idiopathic osteoarthritis.

## Introduction

Osteoarthritis (OA) is the most common form of arthritis and is among the leading causes of disability throughout the world. It is a multifactorial disorder with multiple risk factors contributing to its onset and progression, such as age, genes, hormones, and lifestyle [1]. The most common form of OA is that of the hand [2].

Evidence of a genetic influence on OA originates from various studies, including those on family history and familial clustering, twin studies, and examination of rare monogenic disorders. Estimates of the heritability of OA have ranged from 27% to 65% [3-5]. A number of candidate genes have been implicated by association studies in the pathology of OA. Among them are the genes for the vitamin D receptor [6], collagen type II [7], and the estrogen receptor-α [8]. However, these genes can explain only a small part of the genetic component. Up to now, the pathogenesis of OA is poorly understood. Anatomical, physiological, and immunological processes seem to be involved. With a disease as complex in etiology as OA, all of the possible structural and functional susceptibilities make it hard to make an educated guess about the involvement of a particular gene. In such a situation, the analysis of a rare monogenic disorder with an overlapping phenotype may give a clue to the right gene or pathway.

Recently, we identified the genetic defect in patients with generalized arterial calcification of infancy (MIM#208000) [9]. In addition to calcifications of great and medium-sized arteries, periarticular calcifications and inflammation of the wrists and ankles were observed in many patients [10,11]. We found numerous disabling mutations in the gene coding for ectonucleotide pyrophosphatase/phosphodiesterase 1 (ENPP1) in these patients [9,12]. This enzyme regulates soft-tissue calcification and bone mineralization by generating inorganic pyrophosphate (PP_i_), a solute that triggers cell differentiation and serves as an essential physiological inhibitor of hydroxyapatite deposition [13]. In the corresponding mouse model, the 'tiptoe walking' (or *ttw*/*ttw*) mouse, ectopic ossification of the joints and the ligament of the spine are the striking features, while spontaneous arterial calcification seems to be of minor relevance for the health of the mice [14,15]. The phenotypic consequences of *ENPP1 *mutations in men and mice suggest that genetic variability of ENPP1 activity may contribute to common forms of articular disorders [16].

In this study, we investigated variants of the *ENPP1 *gene to analyze whether they have effects on the development of hand OA in a large sample of Caucasian nuclear families. We focused our attention on a relatively isolated population in southern Russia, the Chuvashians. They live in small villages in the Chuvasha and Bashkirostan autonomies of the Russian Federation. They have lived there for generations, their family structures are stable, and their environmental conditions have been relatively constant. Our data support the hypothesis that *ENPP1 *is a major candidate gene for OA susceptibility.

## Materials and methods

### Population sample

We studied 574 adults, 294 men and 280 women, ranging between 18 and 90 years of age. They belonged to 126 two- to four-generation pedigrees. The subjects were uniformly distributed with respect to age between 20 and 70 years. The pedigrees comprised 3 to 14 persons. The studied individuals were all Chuvashians (Caucasians) from small villages in the Chuvasha and Bashkirostan autonomies of the Russian Federation. Their population is demographically stable and they have lived there for centuries. The environmental conditions, particularly dietary influences, have been relatively constant and genetic flow has been minimal [17]. Further details on this relatively isolated population, and our contacts with them, are given elsewhere [18].

Our genetic field work in Chuvasha consisted of a complete history, physical examination, and health questionnaire conducted by a native speaker. All diagnostic measures were in compliance with the Helsinki Declaration. Written, informed consent was obtained after due approval by the local ethics committee. Plain posteroanterior radiographs of both hands were taken from each study participant, with the x-ray source located 60 cm above and using a standard radiographic technique [18]. Digital images were created from all radiographs. The extent of OA development was evaluated for each of the 14 joints of each hand separately, in accordance with the grading system of Kellgren and Lawrence [19]. The OA evaluation was based on radiographic changes, such as presence of osteophytes, joint-space narrowing, subchondral sclerosis, lateral deformity, or cortical collapse. Development of OA at each joint was graded from 0 to 4. Since the OA scores for individual joints are intercorrelated, the total individual OA score was obtained from principal component analysis of grade sums for all assessed joints. First factor scores (FS1-OAs) were then used in further analyses as a characteristic of hand OA. We recently described the method in detail elsewhere [20]. To assess the reproducibility of our basic phenotype, the evaluations of each trait was performed twice on 50 randomly chosen radiographs by the same investigator 10 days apart. The κ statistic showed high intraobserver reproducibility for the Kellgren and Lawrence (K–L) score (κ = 0.87; *P *< 0.01) and was in good agreement with that found in other studies on a similar subject [21].

### Genotyping, quality check, and haplotype reconstruction

DNA was prepared from peripheral blood lymphocytes by standard techniques. Single-nucleotide polymorphisms (SNPs) were previously identified when sequencing the *ENPP1 *gene in 23 unrelated patients with generalized arterial calcification of infancy, together with their parents and a number of control individuals [9,12]. All but the missense mutation R774C can also be found in the public databases [22] and may be referred to by their rs numbers. Genotyping was performed by Pyrosequencing™ on the PSQ™ HS 96A System (Biotage AB, Uppsala, Sweden). Primer sequences for the assays are available upon request. Amplification conditions were standard as specified by the supplier. Controls were included to exclude mix-ups and other errors during genotyping. Thus, each plate contained a well with DNA-free reaction mix to detect contamination with DNA. Another well contained a dedicated DNA, which was expected to yield identical genotypes for all plates genotyped for a given genetic variant.

We identified three new short tandem repeat (STR) markers at the *ENPP1 *locus at 6q, one of the (tcct)_n _tetranucleotide repeat type (M06NR1A) and two of the common (ca)_n _dinucleotide repeat type (M06NR2A and M06NR3A), and genotyped them in all samples in addition to the SNPs. The exact position in base pairs of the three STR markers in contig NM_006208.1 is at 8776828ff base pairs for M06NR1A, 8862795ff base pairs for M06NR2A, and 8911166ff for M06NR3A. For each marker, 6 ng of genomic DNA was amplified in a 10 μl reaction volume on an MJ PTC 225 Tetrad Cycler. The PCR mix contained 0.53 μM each primer (sequences are available upon request), 0.1 μM each dNTP, 0.5 U *Taq *polymerase, 1 × reaction buffer with 1.5 mM MgCl_2_. The forward primers were labelled at their 5' ends with FAM. Genotypes were determined on a MegaBACE 1000 automated sequencer (Amersham Biosciences, Freiburg, Germany). For allele calling, the proprietary Genetic Profiler software version 1.5 from Amersham Biosciences was used.

Genotyping data were checked for Mendelian errors with the PedCheck program [23]. In two cases, implausible Mendelian errors were detected, and the two probands were excluded from further analysis. It was also tested whether the observed recombination rates between the markers were in accordance with their distance. We did not observe any hint of genotyping errors.

Haplotypes for all individuals were determined by using the program Genehunter version 2.1 [24]. SNPs were arranged according to their location on the chromosome in the following order: rs1800949, rs858342, rs1044498 (K173Q), R774C.

### Statistical and genetic analyses

Data analysis was carried out in two steps. We first used variance component analysis as implemented in the FISHER program [25] to assess the contribution of genetic and common environmental factors in families to OA variation as compared to the influence of potential covariates, such as sex, age, body weight, and height. We recently described the method in detail elsewhere [26]. Briefly, the program uses a maximum likelihood ratio test (LRT) as a model-fitting technique. It simultaneously assesses the contribution of each of the potential covariates (sex, age, etc.) and the contributions of the putative sources of the familial variation, namely, additive genetic effect (V_AD_) and common environment shared by parents (V_SP_), by siblings (V_SB_), and by all members of nuclear pedigrees/household (V_HS_). First factor scores (FS1-OAs) were adjusted for all significant covariates, that is, age and sex, before proceeding to the second step of the analysis.

As a second step, we then employed transmission disequilibrium tests (TDTs) to detect an association between hand OA as a quantitative trait (FS1-OA) and selected DNA marker alleles. Three TDT-like tests were carried out for each pair of dependent variable and specific genetic marker or haplotype. They included the orthogonal test (OT) proposed by Abecasis and colleagues [27] and implemented in the quantitative transmission disequilibrium test (QTDT) program, the family-based association test (FBAT) proposed by Horvath and colleagues [28] and implemented in the FBAT program, and the extreme offspring design *t*-test (EOT) proposed by Malkin and colleagues [29] and implemented in the MAN-6 package. The OT is the maximum likelihood test based on orthogonal decomposition of genotype scores. The significance of the additive impact of within-family genotype score on the phenotype is tested in the OT. The FBAT examines a similar hypothesis – that the phenotype is independent of a specific genotype – but by different statistical algorithms. The EOT extends the ideology of Allison's Q3 test [30] by optimal choice of the extreme offspring in each nuclear family.

The simultaneous application of different tests requires interpretation and pooling together of the various *P *values obtained in testing the same pedigree sample. We computed the combined *P *value, the probability of erroneous rejection of the general null hypothesis of no linkage disequilibrium, which unites certain null hypotheses for separate tests. The combined test can be constructed in two ways: one (A) using the asymptotic χ^2 ^distribution, and the other (B) using the simulated joint distribution of three separate test values.

#### (A) Combined test by asymptotic χ^2 ^distribution

The orthogonal test is the likelihood ratio test with χ^2 ^distribution. The FBAT and EOT use normal and Student's distributions, respectively. Theoretically, the square of FBAT and asymptotically the square of EOT are also distributed as χ^2^. If the above TDTs are considered as independent investigations, the corresponding *P *values will also be independent and the sum of three tests is distributed as χ^2^. This distribution then can be used to obtain an overall *P *value for all three tests as a combined probability of all three null hypotheses together [31]. However, if the three tests are not independent, the distribution of the sum can deviate from χ^2 ^to an extent depending on the overlap of the areas of false-positive results in the separate tests.

#### (B) Simulated joint distribution of three separate test values

We generated 20,000 simulation replicates to generate the joint three-dimensional null distribution for three tests. We used the pedigree structure of our sample and the trait inheritance model exhibiting the observed familial correlations, but assuming no effect of the tested marker on the trait variation. Then for each *P *value triad α_1_<α_2_<α_3_, the probability can be found that the following condition is true: all three separate tests have *P *values not greater than α_3_, at least two of them have *P *values not greater than α_2_, and at least one of them has a *P *value less than α_1_. If we now use the defined condition as a rejection of the general null hypothesis of no linkage disequilibrium, then the described probability is the probability of erroneous rejection of the general null hypothesis based on the simultaneous combination of separate test results. The obtained value can be treated as a combined *P *value, accounting for the extent of overlap of the areas of false-positive results in different TDTs on a sample of given structure.

For the multiallelic STR markers, we examined only those alleles for which the dichotomy factorization produced more than 40 informative nuclear families in our sample. This minimum-number-of-nuclear-families criterion was introduced after the simulation study, investigating the dependence of the ratio of type I error to the test power on the number of informative families. To account for the number of tested alleles for each STR marker, the Bonferroni correction was made. It was performed for each STR marker, using for the separately tested allele the combined *P *value obtained as described in the previous paragraph under (A) and (B). The STR markers presented with 6 to 13 alleles. To facilitate analysis, low-frequency alleles were combined and the three most frequent alleles of each marker were used as they presented.

We also applied the pedigree disequilibrium test (PDT). The PDT examines the trait inheritance under the assumption that the marker locus itself is the gene controlling a part of the trait variation. The distribution of residuals is modeled as an *n*-dimensional normal with familiar partial correlation coefficients estimated as parameters. The LRT is used to reject the null hypothesis that all marker genotypes exhibit the same mean trait value. Here the complete pedigree data are analyzed instead of only members of informative nuclear families as in TDT. This test is very sensitive to disequilibrium. Our comparison of power of the PDT to detect the simulated linkage disequilibrium against TDTs (I Malkin and G Livshits, Accounting for the quantitative trait variance shared by family members significantly improves the power of linkage disequilibrium tests; under review) in the present sample for markers influencing only a small portion (<0.10) of the total trait variance consistently showed substantial superiority of the PDT.

## Results

### Characteristics of the study sample and heritability of OA

**Figure 1:**
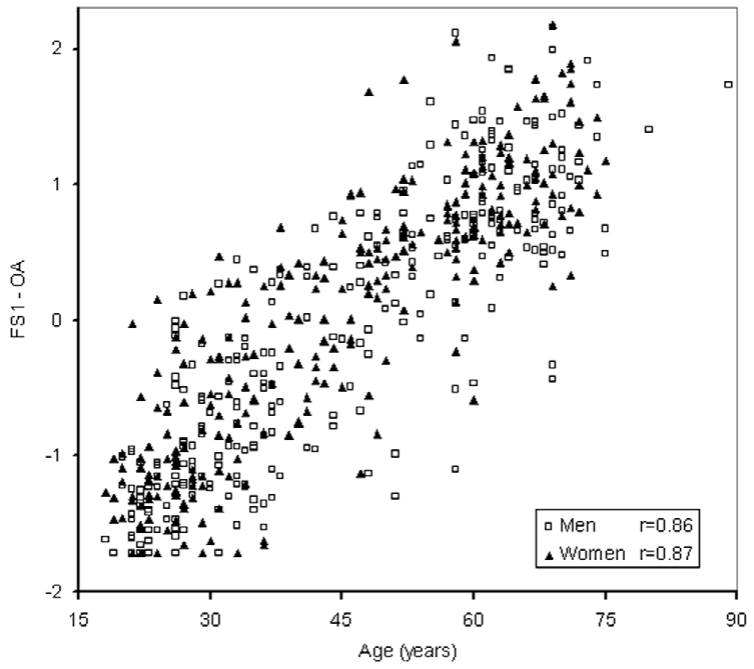
Age-dependence of osteoarthritis of the hand in men and women in the Chuvashian population sample. FS1-OA is the first factor score obtained from the principal component analysis of OA (osteoarthritis). The regression coefficients were calculated using the statistical package FISHER [25].

Table 1 gives the demographic data and OA measures for the subjects. The data are presented according to 15-year age ranges. The agewise distribution of the subjects was fairly uniform. The men were larger than the women. Body mass index (BMI) increased until middle age and then either decreased (men) or remained constant (women). Both genders were nearly equally affected by OA, in a strongly age-dependent manner. As expected from many previous studies, a practically linear increase of the FS1-OA was seen in both the male and the female cohorts after the age of 30 years (Fig. 1).

Variance decomposition analysis was performed to estimate the contribution of genetic factors to the interindividual FS1-OA variation in comparison with the effect of the potential covariates (Table 2). The age effect was highly significant in both genders, and the corresponding regression parameter estimates were in good agreement with those obtained by the least mean square method as used in Fig. 1. The correlation with age was not sex-dependent and explained 74.3% of the total variation. However, sex differences were significant at the intercept of the regression equation. The body weight and height of the individual exerted negligible effect on variation in OA of the hand. Of the familial influences, putative genetic effects were statistically a most significant factor by the LRT (*P *< 0.001). Nearly 25% of the age-adjusted FS1-OA variation was attributable to genetic factors. Common environment shared by spouses also made a significant contribution (approximately 13%) to FS1-OA variation. Constraining this effect to zero was rejected by the LRT (*P *< 0.05).

### Family-based association study with DNA markers of the *ENPP1* locus

**Figure 2:**
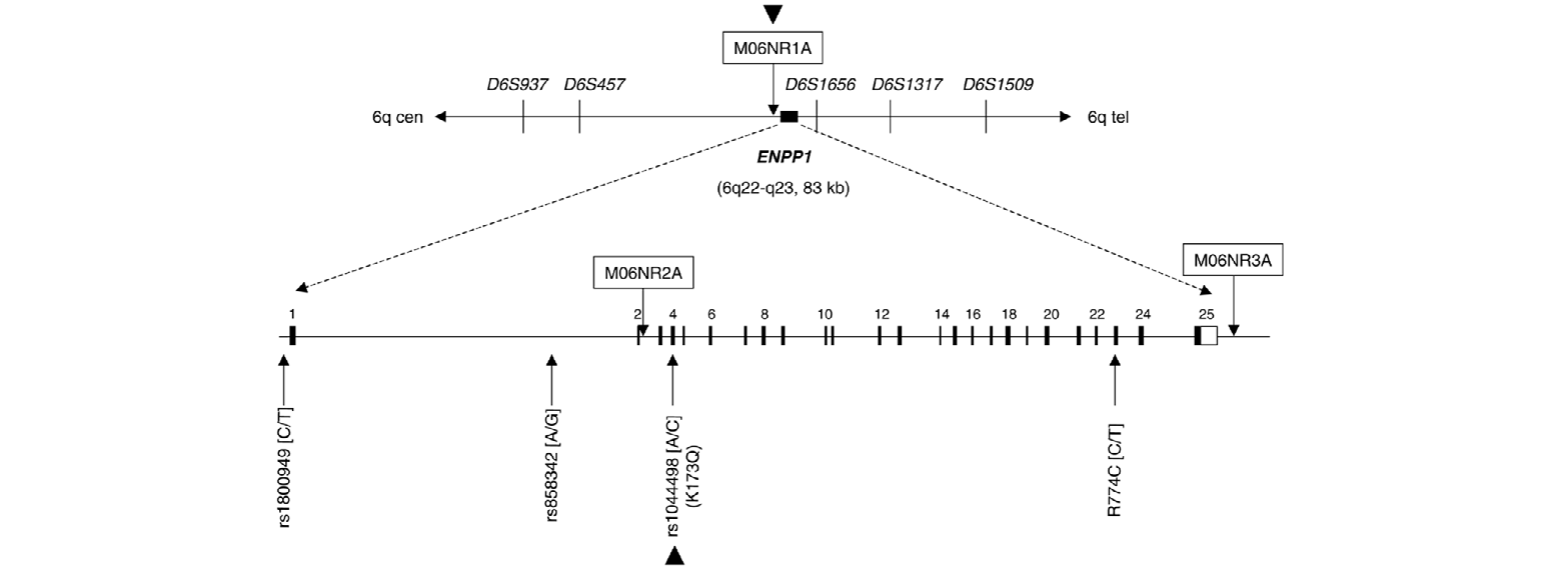
Map of the *ENPP1 *locus. The 25 *ENPP1 *exons (boxes) are numbered from left to right according to the direction of transcription. The filled boxes constitute the coding region. The open box represents the long 3' untranslated region. Vertical arrows point to the polymorphic sites analyzed in this study. M06NR1A is located some 46 kilobase pairs (kb) upstream of the promoter. The other intermarker distances may be taken from the graph, which is drawn to scale. DNA markers with alleles that were found to be significantly associated with hand OA (see Tables 3 and 4) are marked by arrowheads.

**Figure 3:**
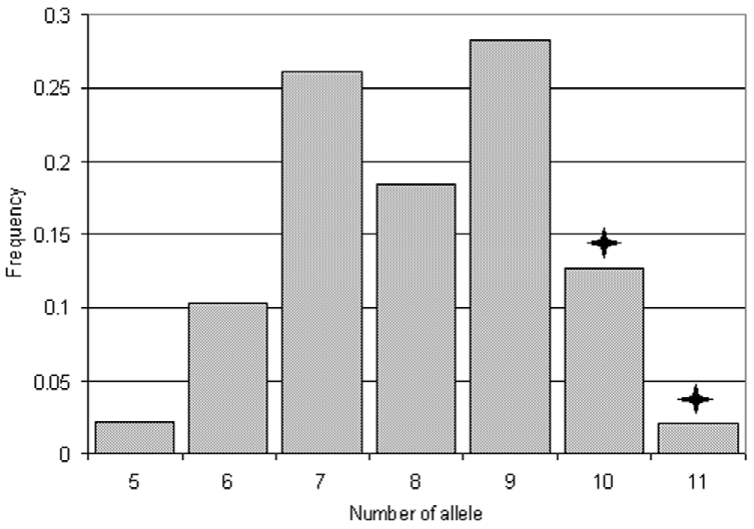
Allele frequencies of tetranucleotide repeat short tandem repeat marker M06NR1A. The strongest signals of association with hand osteoarthritis were obtained with alleles 10 and 11 (marked by star symbols).

We selected three STR and four SNP markers of the *ENPP1 *locus to obtain a fairly complete coverage of the gene region (Fig. 2). Analysis of linkage disequilibrium between each of the three STR markers on the one hand and the SNPs on the other revealed sufficient coverage of the entire *ENPP1 *locus to detect a functional SNP via linkage disequilibrium by the three STRs (data not shown). The markers were genotyped in all individuals of the population sample. To test whether particular alleles of any of the markers were significantly associated with age-adjusted FS1-OA, we used three different TDT-like tests and PDT (Table 3). For the three TDT-like tests, we also estimated the combined probabilities of the null hypothesis rejections, assuming either that the three tests (A) were  or (B) were not independent. Using the different tests and combined analyses, we were able to demonstrate a number of significant (*P *< 0.05) or even highly significant (*P *< 0.001) associations between the rare-allele pool of M06NR1A ('allele' 4F), the C-allele of K173Q, and various haplotypes of two or three adjacent SNPs (Table 3). For the haplotypes, the combined three test *P *values ranged from 0.0082 to 0.000018, and from 0.020 to 0.0006 for the A and B types of computation, respectively. When the rare-allele pool of M06NR1A, alleles 5 + 6 + 10 + 11 (Fig. 3) was split into its components, allele 10 and the combination of the adjacent alleles 10 and 11 showed the strongest association signals with A-type combined *P *values, as low as 0.0001 and 0.000004, respectively (Table 4). Even after Bonferroni correction for the number of tested alleles per marker, all *P *values remained significant. PDT results were generally in agreement with the TDT results (Table 4). Even though the results of three tests for particular haplotypes were no longer significant, the others achieved statistical significance, with *P *values between 0.0278 and 0.0002.

## Discussion

In the present and a previous study [4], we have demonstrated a strong genetic component determining OA of the hand in a population sample of Chuvasians. Moreover, our present study provides strong evidence that there is a substantial contribution of *ENPP1 *variants to this genetic component. In a family-based association study using three STR and four SNP markers covering the entire *ENPP1 *locus, we consistently found significant associations between several SNP haplotypes and hand OA as quantified by the Kellgren–Lawrence method [19]. As a single marker, only the C-allele of the K173Q polymorphism was found to be associated with hand OA, though less significantly than inferred haplotypes including K173Q. Thus K173Q itself is unlikely to be the functional variant underlying the association signal. The most impressive association lead was found with the pooled rare alleles of the STR marker M06NR1A, which was associated with a younger age at onset by a mean of about 3.5 years (Table 3). The major contribution to this signal came from the two largest alleles, 10 and 11 (Fig. 3). M06NR1A is located some 46 kb upstream of the gene, suggesting that the functionally relevant variant(s) may regulate the expression of *ENPP1*. We do not believe that the STR alleles themselves regulate the expression level; rather, they are likely to tag a regulatory haplotype.

Our reason for studying the influence of *ENPP1 *variants on the development of OA came from the observation that patients with generalized arterial calcification of infancy often showed joint cartilage mineralization as well. The main function of ENPP1 in the extracellular matrix is to generate PP_i _from nucleoside triphosphates, indicating that extracellular PP_i_, which suppresses hydroxyapatite crystal growth, might be the key factor in the regulation of numerous mineralization processes [11,16].

Two other genes are also involved in the regulation of extracellular PP_i_. One is *ANKH*, coding for a multipass transmembrane protein that is thought to transport PP_i _from inside to outside the cell [32]. The other gene is *ALPL*, which codes for tissue nonspecific alkaline phosphatase (TNSALP). This enzyme directly antagonises the PP_i_-generating function of ENPP1 by cleaving PP_i _into phosphate [33]. Interestingly, mutations of *ANKH *have been identified in patients with familial articular chondrocalcinosis type 2 (MIM#118600) [34-36]. The deposition of calcium-containing crystals in articular cartilage observed in these families is a common finding that is frequently associated with advanced OA. In contrast to generalized arterial calcification of infancy, in which hydroxyapatite crystals are formed, calcium pyrophosphate dihydrate crystal deposition is observed in these patients due to matrix supersaturation with PP_i_. Nevertheless, both diseases underline that a concerted regulation of PP_i _by the three genes mentioned is critical to avoid mineralization disorders [37]. Thereby the direct antagonistic action of TNSALP against ENPP1 opens new avenues to treatment of such disorders, by the use of either TNSALP or ENPP1 inhibitors as drugs [38].

## Conclusion

The association that we have found with marker alleles at the *ENPP1 *locus explains up to 3.2% of the population variation of FS1-OA. The contribution of the whole PP_i _pathway to the genetic component of the disease development may be considerably larger. To better understand the role of PP_i _in OA, studies are in progress to analyze the influence of the genetic variation of all three PP_i _regulator genes on the variation of the disease phenotype.

## Abbreviations

BMI = body mass index; ENPP1 = ectonucleotide pyrophosphatase/phosphodiesterase 1; EOT = extreme offspring design *t*-test; FBAT = family-based association test; FS1-OA = first factor score obtained from principal component analysis of OA; kb = kilobases; K–L = Kellgren and Lawrence; LRT = likelihood ratio test; OA = osteoarthritis; OT = orthogonal test; PDT = pedigree disequilibrium test; PP_i _= inorganic pyrophosphate; QTDT = quantitative transmission disequilibrium test; SNP = single-nucleotide polymorphism; STR = short tandem repeat; TDT = transmission disequilibrium test; TNSALP = tissue nonspecific alkaline phosphatase.

## Competing interests

The authors declare that they have no competing interests.

## Authors' contributions

ES performed most of the genotyping and contributed to the interpretation of the data. IM developed software for the statistical analysis of the data, performed the association tests, and contributed substantially to the first draft of the manuscript. SD independently analyzed the data to confirm the results. Assessment of osteoarthritis from hand radiographs was performed by LK. NR established the new microsatellite markers and supervised the allele calling. Field work in Russia was organized by EK. MT designed the pyrosequencing assays for SNP typing. FR suggested testing the candidate gene and contributed to the manuscript. PN and GL together designed the study, supervised and directed the whole project, and wrote the manuscript. All authors read and approved the final manuscript.

## Figures and Tables

**Table 1:** Basic descriptive statistics of the studied sample (*N *= 574) of Chuvasians

Trait, by age group (years)	Males					Females				
	
	Valid *n*	Mean	Minimum	Maximum	SD	Valid *n*	Mean	Minimum	Maximum	SD
Age (years)										
<30	74	24.716	18	29	2.855	70	24.429	18	29	2.942
30–44	75	35.507	30	44	3.786	65	36.123	30	44	4.502
45–59	56	52.696	45	59	4.164	70	52.171	45	59	4.619
≥60	89	66.045	60	89	5.054	75	66.253	60	90	5.149
Weight (kg)										
<30	74	62.009	48.0	81.6	7.634	69	52.199	35.5	86.5	9.601
30–44	75	66.741	46.2	98.1	10.887	65	61.711	42.3	95.8	12.501
45–59	56	68.230	49.9	100.1	12.670	69	64.691	46.3	92.3	10.893
≥60	88	62.640	41.1	94.2	12.122	71	63.632	38.1	101.3	13.777
Height (m)										
<30	74	1.692	1.525	1.880	0.064	69	1.571	1.466	1.775	0.062
30–44	75	1.682	1.507	1.894	0.074	64	1.564	1.457	1.694	0.045
45–59	56	1.652	1.534	1.77	0.051	70	1.538	1.424	1.655	0.049
≥60	88	1.620	1.483	1.727	0.057	71	1.502	1.389	1.654	0.052
BMI (kg/m^2^)										
<30	74	21.661	17.28	28.69	2.432	69	21.074	15.78	31.23	3.136
30–44	75	23.509	18.86	32.46	2.925	64	25.141	17.65	39.77	5.019
45–59	56	24.942	18.875	36.225	4.086	69	27.332	19.123	36.880	4.237
≥60	88	23.813	16.28	33.30	3.983	71	28.108	17.63	43.25	5.357
OA score^a ^(K–L score)										
<30	74	7.321	0	24	5.838	70	7.943	0	24	5.728
30–44	74	14.108	0	32	7.526	64	16.516	0	30	7.229
45–59	56	26.932	6	49	8.802	70	28.914	9	49	7.001
≥60	89	34.870	16	51	6.762	72	35.834	14	51	6.271
FS1-OA^b^										
<30	74	-1.119	-1.707	0.200	0.466	70	-1.066	-1.707	0.214	0.465
30–44	74	-0.579	-1.707	0.784	0.596	64	-0.384	-1.707	0.709	0.574
45–59	56	0.408	-1.287	2.144	0.682	70	0.565	-1.122	2.081	0.534
≥60	87	1.002	-0.446	2.194	0.506	71	1.062	-0.577	2.209	0.459

^a^Observed total score for 14 joints, following Kellgren–Lawrence (K–L) atlas [19]. The scores for left and right hand were averaged. ^b^Standardized factor score that resulted from principal component analysis of 28 joints of both hands. BMI, body mass index; FS1-OA, first factor score obtained from principal component analysis of osteoarthritis (OA); SD, standard deviation.

**Table 2:** Variance component analysis of FS1-OA variation in Chuvashian pedigrees

Parameter	Estimate ± SE
*Regression coefficients*	
Intercept	
α_m_	0.0000^a^
α_f_	0.0633 ± 0.0294
Age effect	
β1_m_	0.0519 ± 0.0011
β1_f_	0.0519^b^
Stature effect	
β2_m_	[0]
β2_f_	[0]
Body weight effect	
β3_m_	[0]
β3_f_	[0]
*Variance components*	
V_AD_	0.0654 ± 0.0208 (24.43%)
V_SP_	0.0355 ± 0.0176 (13.26%)
V_HS_	[0]
V_SB_	[0]
V_RS_	0.1668 ± 0.0219 (62.31%)
*LRT*^*c*^	
χ^2^	9.88
df	7
*P*	0.84

Parameter estimates and corresponding asymptotic standard errors (SEs) are provided for the best fitting and most parsimonious genetic model. [ ] Parameter was fixed at the indicated value. α_m _and α_f _are sex-specific intercepts (m, males; f, females). β_m _and β_f _are sex-specific slopes. ^a^Parameter estimate reached the boundary. ^b^Parameter was fixed to be equal to the previous. ^*c*^LRT for comparison of the most parsimonious and general model where all parameters were estimated. df, degrees of freedom; FS1-OA, first factor score obtained from principal component analysis of OA; LRT, likelihood ratio test; V, variation, due to additive genetic effect (V_AD_) or to common environment shared by parents (V_SP_), by siblings (V_SB_), or by all members of nuclear pedigrees/household (V_HS_).

**Table 3:** Tests of association between *ENPP1 *polymorphisms and osteoarthritis (OA) of hand joints

Marker	Allele/haplotype	Freq.	*P *values of TDT			Combined *P *values for 3 tests^a^		Bonferroni correction		*n *inf.	*n *test	Pedigree-based disequilibrium test	
			
			FBAT	QTDT^b^	EOT	A	B	A	B			*P*	Δ (years)
M06NR1A	4F^c^	0.27	0.0001	1.0e-5	0.0009	<1.0e-6	<5.0e-5	<4.0e-6	<2.0e-5	84	4	0.0002	-3.5
K173Q	1	0.11	0.0129	0.0347	0.0403	0.0020	0.0104	0.0020	0.0104	48	1	0.0196	1.9
_XXX^d^	_221	0.66	0.0147	0.0174	0.0043	0.0002	0.0033	0.0006	0.0099	88	3	0.3430	-1.3
_XXX	_211	0.10	0.0148	0.0373	0.0096	0.0007	0.0074	0.0021	0.0220	39	3	0.0110	2.3
_XX_	_22_	0.67	0.0046	0.0050	0.0030	1.8e-5	0.0006	0.0001	0.0018	89	3	0.1827	-1.7
_XX_	_21_	0.10	0.0114	0.0378	0.0091	0.0005	0.0062	0.0016	0.0185	40	3	0.0114	2.3
__XX	__11	0.12	0.0426	0.0509	0.0426	0.0074	0.0177	0.0147	0.0351	47	2	0.0278	2.1
__XX	__21	0.85	0.0362	0.0515	0.0576	0.0082	0.0205	0.0164	0.0406	58	2	0.1574	-1.6

Osteoarthritis scores were measured by the Kellgren–Lawrence method [19]. K–L scores of 28 joints on both hands, which were used as primary phenotype. The primary phenotype was subjected to principal component analysis resulting in first factor scores (FS1-OAs). After being adjusted for age, FS1-OA was used as a quantitative trait for the association tests. ^a^Combined *P *value using χ^2 ^(A) and simulated three-dimensional null distribution (B). ^b^Column presents orthogonal test, which uses parent trait values as covariates. ^c^F is an artificial 'allele' combining all rare alleles but not the three most frequent ones. ^d^XXXX is the haplotype of the four SNPs (rs1800949, rs858342, K173Q, and R774C). The minor allele frequencies of the four individual SNPs in the study population are 0.24 for the T-allele (2) of rs1800949, 0.21 for the G-allele (1) of rs858342, 0.11 for the C-allele (1) of K173Q, and 0.03 for the T-allele (2) of R774C. Δ, mean difference between the allele carriers and other individuals in age at onset of the disorder; EOT = extreme offspring design *t*-test; FBAT, family-based association test; Freq., frequency; *n *inf., number of informative families for TDT in the sample; *n *test, number of tests performed for the marker (alleles with *n *inf. >40); QTDT, quantitative transmission disequilibrium test; TDT, transmission disequilibrium test.

**Table 4:** Reanalysis of M06NR1A 'allele' 4F

Allele	Freq.	*P *values of TDT			Combined *P *value for 3 tests^a^		*n *inf.	Pedigree disequilibrium test	
				
		FBAT	QTDT^b^	*EOT*	A	B		*P*	Δ (years)
10	0.13	0.0051	0.0054	0.0083	0.0001	0.0016	59	0.0151	-2.6
10 + 11^c^	0.15	0.0019	0.0015	0.0043	4.0e-6	0.0004	63	0.0060	-2.9
5 + 6^c^	0.13	0.0622	0.0216	0.0490	0.0055	0.0181	49	0.1326	-1.6

The artificial 'allele' 4F combines all rare alleles but not the three most frequent alleles. ^a^Combined *P *value using χ^2 ^(A) and simulated three-dimensional null distribution (B). ^b^Column presents orthogonal test, which uses parent trait values as covariates. ^c^Artificial alleles including pairs of adjacent alleles. Δ, mean difference between the allele carriers and other individuals in age at onset of the disorder; EOT = extreme offspring design *t*-test; FBAT, family-based association test; Freq., frequency; *n *inf., number of informative families for TDT in the sample; QTDT = quantitative transmission disequilibrium test; TDT = transmission disequilibrium test.
